# Evolutionary Capacitance and Control of Protein Stability in Protein-Protein Interaction Networks

**DOI:** 10.1371/journal.pcbi.1003023

**Published:** 2013-04-04

**Authors:** Purushottam D. Dixit, Sergei Maslov

**Affiliations:** 1 Biology, Brookhaven National Laboratory, Upton, New York, United States of America; 2 Physics and Astronomy, Stony Brook University, Stony Brook, New York, United States of America; 3 Laufer Center for Physical and Quantitative Biology, Stony Brook University, Stony Brook, New York, United States of America; University of Wyoming, United States of America

## Abstract

In addition to their biological function, protein complexes reduce the exposure of the constituent proteins to the risk of undesired oligomerization by reducing the concentration of the free monomeric state. We interpret this reduced risk as a stabilization of the functional state of the protein. We estimate that protein-protein interactions can account for 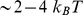 of additional stabilization; a substantial contribution to intrinsic stability. We hypothesize that proteins in the interaction network act as *evolutionary capacitors* which allows their binding partners to explore regions of the sequence space which correspond to less stable proteins. In the interaction network of baker's yeast, we find that statistically proteins that receive higher energetic benefits from the interaction network are more likely to misfold. A simplified fitness landscape wherein the fitness of an organism is inversely proportional to the total concentration of unfolded proteins provides an evolutionary justification for the proposed trends. We conclude by outlining clear biophysical experiments to test our predictions.

## Author Summary

The folded form of proteins is only marginally stable *in vivo* and constantly faces the risk of aggregation, unfolding/misfolding, and other aberrant interactions. For most proteins, the folded form is also the functionally relevant one and forces of natural selection strongly modulate its stability. *In vivo*, proteins interact with each other on a genome-wide scale. Usually, the interaction of a protein and its binding partners requires both the proteins to be in the folded form and as a result, the interactions tend to shift the population of a protein towards the folded form. Consequently, protein-protein interactions interfere with the evolution of protein stability. Here, we present empirical evidence and theoretical justification for proteins' ability to stabilize the folded form of their interaction partners and allow them to explore the region of the sequence space that corresponds to proteins with less stable structure. We argue that the ‘evolutionary capacitance’ – previously thought to be a property of the chaperone HSP90, a special class of proteins – is a property of all proteins, albeit to a different degree.

## Introduction

The toxicity due to protein misfolding and aggregation has a considerable effect on the viability of living organisms [1]–. Consequently, cells are under strong selection pressure to evolve thermodynamically stable [6] and aggregation-free protein sequences [7]. The internal region of stable proteins has a tightly packed core of hydrophobic residues. A mutation in the core may disrupt the entire protein structure. Consequently, the core residues are strongly conserved [8], [9]. In contrast, mutations on the surface contribute weakly to the thermodynamic stability of proteins [10] yet surfaces show significant level of conservation [11] owing to protein-protein interactions.

Recent high throughput experiments have established that proteins interact with each other on a genome-wide scale [12]. Such ‘small world’ networks are thought to facilitate biological signaling and ensure that cells remain robust even after a random failure of some of its components [13]. It is thought that evolutionarily, multi-protein complexes are favored over larger size of individual proteins [14] since large proteins are difficult to fold and expensive to synthesize while small interacting proteins can fold independently and then efficiently assemble into large complexes. Individual interaction between proteins can give rise to cooperativity and allostery which results in a finer control over the functional task the protein complex performs. Protein-protein interactions (PPI) are also thought to prevent protein aggregation [15], [16]. Lastly, many proteins can perform promiscuous function in that they can partake in multiple protein complexes. Interestingly, proteins in higher organisms are involved in more interactions and form larger protein complexes compared to more primitive life forms [17].

**Figure 1:**
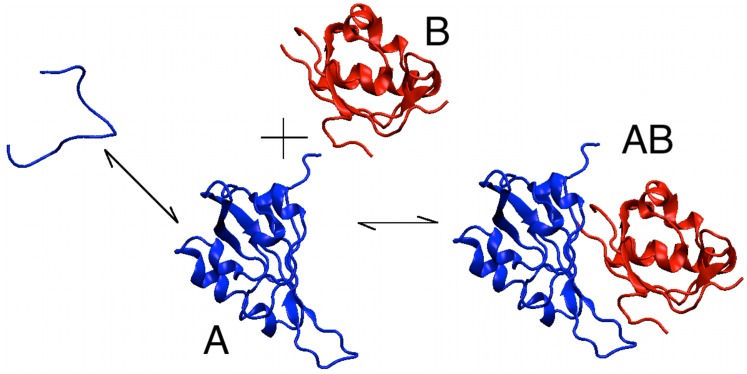
The equilibrium between the folded state of protein A (blue protein) and its unfolded/insoluble state (blue coil) is affected by the interactions of the folded state with its interaction partner B (red). The formation of the AB dimer lowers the population of the unfolded/insoluble state of protein A and effectively stabilizes the folded state.

Here, we hypothesize an additional biophysical advantage for protein-protein interactions. Proteins bound to their interaction partners *effectively* present a lower monomer concentration inside the cell. Since free monomers are susceptible to misfolding/unfolding and toxic oligomerization, interacting proteins may face a reduced risk towards the same. This reduced risk can be interpreted as interaction-induced stabilization 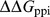 — stabilization due to the protein-protein interaction network — of an otherwise monomeric protein (see Fig. 1 for a cartoon). We propose that by giving proteins an additional stability, each protein in the interaction network acts as an *evolutionary capacitor* [18], [19] in the evolution of its binding partners: proteins are allowed to explore the less stable regions (regions of low intrinsic stability) of the sequence space as long as they are stabilized by their interaction partners. Inversely, unstable proteins are expected to receive significant additional stability from the interaction network.

Below we outline the empirical evidence for our hypothesis and suggest clear biophysical and evolutionary experiments to test it further.

## Results

We present our estimates of the interaction-induced stability 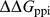 (see Methods) and explore the evolutionary interplay between 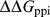 and protein stability 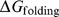 using a simplified fitness model for a toy proteome. We test the predictions of the toy model on the proteome of baker's yeast. The fitness model also sheds light on the interplay between protein stability and protein abundance.

### Interaction-induced stability 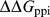 is comparable to inherent stability 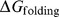


**Figure 2:**
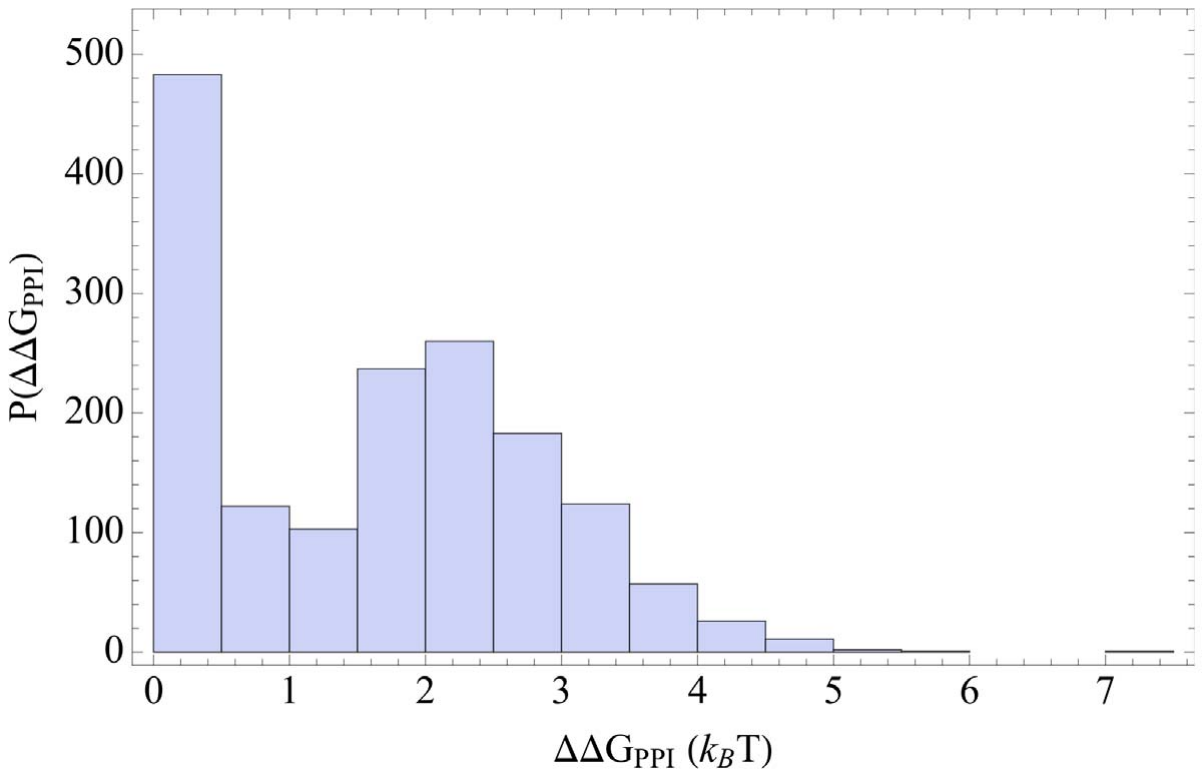
The histogram of estimated PPI-induced stabilities for the yeast cytoplasmic proteome (See main text). While the average stability is 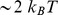, some proteins can receive as much as 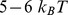 of stability from their binding partners. Note that the peak near 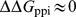 is due to proteins which have no interaction partners and are by definition not stabilized by the PPI network.

Fig. 2 shows the histogram of the estimated interaction-induced stability 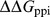 for 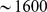 cytoplasmic yeast proteins for whom abundance, interaction, and localization data is available (see Methods for the details of the calculations). Note that the average PPI induced stability is 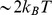 and can be as high as 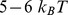. This stabilization is dependent not only on the number of interaction partners of a given protein or the strengths of those interactions but also on the relative abundances of the interaction partners. In fact, the interaction-induced stability of a protein correlates strongly with the relative concentration of its binding partners
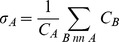
(Spearman 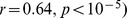. This suggests a plausible mechanism of stabilization of a protein without changing its sequence viz. via adjusting the expression levels of its interaction partners (see Discussion below).

The estimated 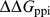 values are of the same order of magnitude as the inherent stabilities of proteins, 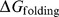 (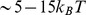) [9]. Given that random mutations are more likely to destabilize proteins [6], we expect protein-protein interactions to act as secondary mechanisms to stabilize proteins and to interfere with the evolution of protein stability.

### Simplified fitness model explores the interplay between 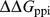 and 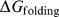


To explore the evolutionary consequences of the interaction-induced stability, we investigate a simplified fitness model of a toy proteome consisting of 15 proteins (see Methods, Text S1, and Table S1). Briefly, the fitness of the cell depends only on the total concentration of unfolded proteins in it [20]. During the course of evolution, each protein acquires random mutations that change either a) its inherent stability 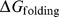 or b) the dissociation constant of its interaction with a randomly selected interaction partner. Even though protein abundance and protein-protein interactions evolve at the same time scale as protein stability, the former are dictated largely by the biological function of the involved proteins. Incorporating the fitness effects of changes in expression levels and interaction partners in our simple model is non-trivial. Thus, in order to specifically probe the relation between stability and interactions, we do not allow proteins to change their abundance and interaction partners.

In the model, the concentration of unfolded proteins and thus the fitness of the proteome depends on the total stability 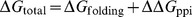 of individual proteins. While random mutations are more likely to make proteins unstable, protein-protein interactions increase the total stability. In the canonical ensemble description of the evolution of fitness [21], the inverse effective population size (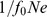), the *evolutionary temperature* quantifies the importance of genetic drift. The *effective* population size modulates the competition between destabilizing random mutations and stabilizing protein-protein interactions.

**Figure 3:**
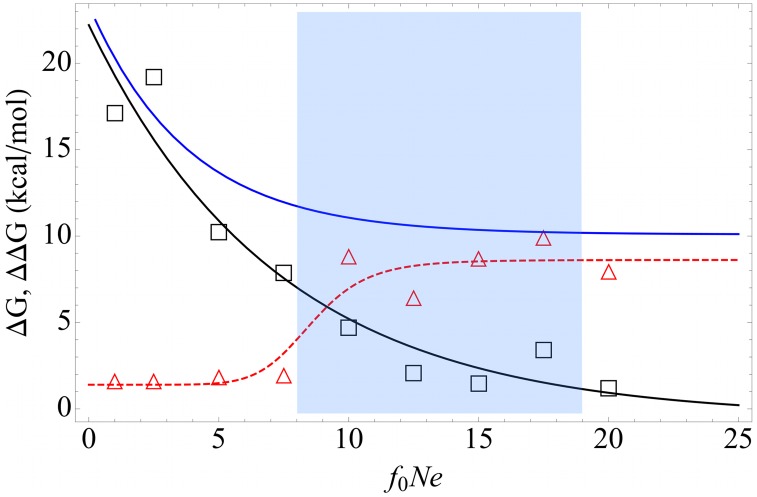
The average of inherent stability 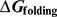 (triangles) and the interaction-induced stability 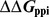 (squares) as a function of effective population size 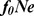 for the toy proteome. The curves are fitted to the data only to highlight trends, blue curve represents the total stability 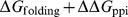. Population size 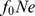 is in arbitrary units. The shaded area roughly represents the region of the red and the black curve that correspond to the empirically observed folding free energies 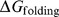 (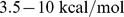) [9] and the estimated interaction-induced free energy 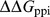 (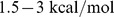).

We find that at higher effective populations, proteins are inherently stable and only the least stable proteins (small 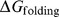) receive high stabilization from the interaction network (high 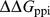). At low effective population, due to genetic drift, proteins are inherently destabilized and protein-protein interactions serve as the primary determinant of the *effective* stability of proteins. Fig. 3 shows the dependence of average inherent stability (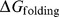), average interaction-induced stability (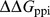), and average total stability (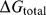) with effective population size. Interestingly, the total stability (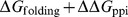) of proteins remains relatively insensitive to changes in population size.

**Figure 4:**
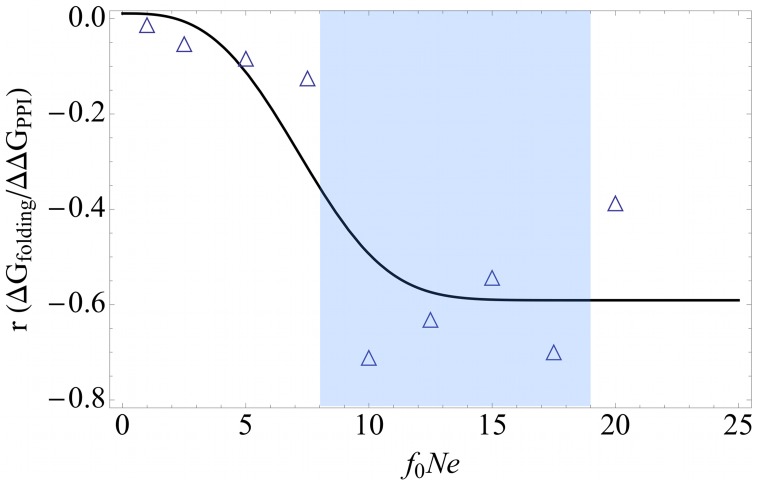
The spearman correlation coefficient between interaction-induced stability 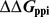 and inherent stability 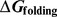 as a function of effective population size 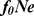 (See supplementary Text S1). Population size is in arbitrary units. The blue region identifies the location of real life proteomes (See Fig. 3).

We observe that the correlation coefficient between the inherent stability 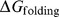 and the interaction-induced stability 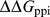 itself varies with the effective population size. Even though its magnitude decreases, interaction-induced stability becomes more and more correlated with inherent stability as population size increases (See Fig. 4). In real life organisms, interaction-induced stability acts on a need basis for proteins and serve as a secondary stabilization mechanism. In the drift-dominated regime, which is unlikely to be realized in real life organisms (except probably in parasitic microbes with low population sizes), interaction-induced stability becomes the dominant player in the evolution of total stability of proteins [17]. We next examine if this prediction from the toy model holds for real organisms.

### Induced stability correlates with aggregation propensity

Proteome-wide information about the inherent stability of proteins 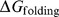 is currently unavailable. Previously, *in silico* estimates of protein aggregation propensity have been used as proxy for protein stability [22], [23]. We use the TANGO [24] algorithm to estimate protein aggregation propensity. It is known that TANGO aggregation propensity correlates strongly and negatively with protein stability [24]. TANGO has been verified extensively with experiments on peptide aggregation [24] and has been previously used to study the evolutionary aspects of protein-protein interactions [22], [25]. Similar analysis for Aggrescan [26] can be found in Text S1 and Table S3. We find that the aggregation propensity 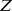 is correlated positively with the interaction-induced stability 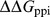 (Spearman 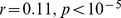). As expected [2], the aggregation propensity 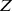 is negatively correlated with protein abundance 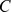 (Spearman 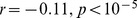). The correlation between 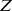 and 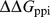 does not depend on this underlying dependence and persists even after controlling for total abundance 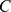 (partial Spearman 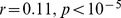) (See Table S2). This result suggests in the proteome of baker's yeast, protein stability correlates negatively with interaction-induced stability.

### Aggregation propensity correlates *principally* with free monomer abundance

The fitness cost of protein aggregation is directly proportional to the amount of aggregate [20]. Thus, the selection forces that make protein sequences aggregation-free act more strongly on highly expressed proteins [1], [2], [22]. Our hypothesis suggests that the proteins that are bound to their interaction partners present a lower concentration of the *free monomeric* state *in vivo* (low 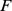) and automatically lower the misfolding/aggregation induced fitness cost, even if highly abundant (high 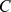). The selection forces to evolve an aggregation-free sequence may be weaker for such proteins. Consequently, the aggregation propensity 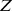 should be principally correlated with the free monomer concentration 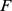 rather than the total abundance 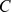.

Indeed, we observe that the estimated monomer concentration 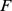 and the aggregation propensity 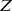 are correlated negatively (Spearman 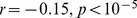). Importantly, this correlation is not an artifact of the underlying correlation between the aggregation propensity and total abundance 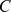 (partial Spearman 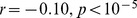). At the same time, the partial correlation coefficient between the aggregation propensity 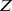 and the total protein abundance 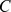 controlling for the estimated monomer concentration 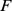 is minimal (partial Spearman 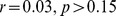). In short, the total free monomer concentration 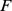 of a protein (rather than 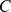, its total abundance) might be a better variable to relate to evolutionary and biophysical constraints on the protein.

### Interacting proteins as evolutionary capacitors

We have thus far shown that a protein's interaction partners can significantly stabilize its folded state and this stabilization interferes with the evolution of the inherent stability of the protein. We now explore the reverse viz. the evolutionary consequences of the ability of each protein to impart stability to its interaction partners.

The concept of evolutionary capacitor has been previously introduced for the heat shock protein HSP90 [18], [19], which is also a molecular chaperone and a highly connected hub in the PPI network (70 interaction partners in the current analysis). An elevated concentration of HSP90 buffers the potentially unstable variation in proteins, which may allow proteins to sample a wider region of the sequence space, which may often lead to functional diversification [27]. Similar to HSP90, each protein in the interaction network has some ability to stabilize its interaction partners to a certain extent. Consequently, we study the evolutionary *capacitance* 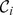 of individual proteins in the context of the interaction network by estimating the effect of protein knockout on ppi-induced stability *in silico*. Proteins with higher evolutionary capacitance are defined as those with the higher cumulative destabilizing effect on the proteome. We write,
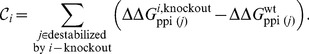
(1)For each protein 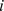, the sum in Eq. 1 is carried out over all proteins 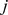 that are destabilized due to its knockout. Here, we assume that the potential of a given protein knockout to generate multiple phenotypes depends on the loss of stability of its interaction partners caused by its knockout. We hypothesize that, similar to unstable proteins requiring HSP90 to fold, the interaction partners of proteins with high capacitance should be unstable. In fact, the capacitance 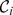 of a protein and the mean aggregation propensity 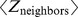 of its interaction partners are strongly correlated (Spearman 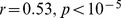). The capacitance 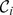 is significantly correlated with 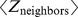 even after controlling for the abundance of the protein (partial spearman 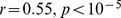) and the number of its interaction partners (partial spearman 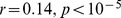). This suggests that a protein needs to be present in sufficient quantity and should interact with a large number of proteins in order to effectively act as a capacitor.

We have presented evidence that all proteins can act as an *evolutionary capacitor*, albeit with variable effectiveness, for their interaction partners. Traditionally, evolutionary capacitors are understood to be chaperones that buffer phenotypic variations by helping misolding-prone proteins fold in a proper structure [19]. Not surprisingly, when we carried out functional term enrichment analysis using gene ontology [28], we found that approximately half of the top 20 capacitors have ‘chaperone’ in their name. The top 20 are also over represented in the chaperone-like molecular function of *protein binding* and *unfolded protein binding* (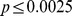) and the biological process of *protein folding* (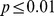). These findings validate our definition of capacitors that were previously identified as chaperones. Interestingly, some of the predicted capacitors do not currently have a protein folding-related functional annotation. These need more experimental investigation (see supplementary File S1 for the list). This suggests that previously identified *evolutionary capacitor* HSP90 may in fact only be one among the broader set of *evolutionary capacitors*. Every protein in the interaction network is an *evolutionary capacitor* for its interaction partners and *evolutionary capacitor* is a quantitative distinction rather than a qualitative one.

## Discussion

Recently, Fernández and Lynch [17] showed that random genetic drift is the chief driving force behind thermodynamically less stable yet densely interacting proteins in higher organisms [17]. Additionally, protein complexes in higher organisms have more members than in lower organisms [14]. Recently, it was observed that a destabilizing mutation in the enzyme DHFR in *E. coli* leads to functional tetramerization of the otherwise monomeric enzyme [29] suggesting that protein-protein interactions can at least partially compensate the effect of protein destabilization. 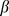 lactoglobulin is an aggregation-prone protein generally found as a dimer. It was shown that the specific interactions responsible for the formation of the dimer considerably reduce the risk of protein aggregation [16]. Ataxin-3 is a protein implicated in polyglutamine expansion diseases wherein the functional interactions of the protein reduce the exposure of its aggregation prone interface and thereby decrease its aggregation propensity [15].

Here, we have quantified the interaction-induced stability on a proteome wide scale and hypothesized that the PPI-induced stabilization is a secondary evolutionary advantage of the PPI network; alleviating the selection pressure on proteins in functional multi-protein complexes to evolve a stable folded. A simple model for the fitness of the proteome provided a fundamental justification for the co-evolution of protein stability and protein-protein interactions and made predictions that were tested on the proteome of baker's yeast. In the model, when the effects of natural selection are weak, proteins acquire stability mainly via protein-protein interactions. At a higher population size — in the absence of genetic drift — proteins are intrinsically stable and protein-protein interactions stabilize only those proteins that fail to evolve inherent stability.

We have also presented evidence that *all* interacting proteins stabilize their binding partners to a certain extent and act as the evolutionary capacitance [19] for their evolution. Interestingly, though some of the top 20 capacitors predicted in this study are known chaperones and are over-represented in GO ontology terms such as *protein binding*, *unfolded protein binding*, and *protein folding*; others do not have any protein folding-related functional annotation and need experimental investigation.

The importance of disordered proteins, especially in the proteomes of higher organisms, cannot be neglected. The proteome of baker's yeast does not have many completely disordered proteins but 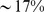 of the amino acids in the proteins of yeast are predicted to be in a disordered state [30] (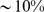 for the proteins considered in this study, see supplementary Text S1 and Fig. S4). Even though the development presented above applied only to an equilibrium between folded and unfolded/misfolded/aggregated protein, it can be easily generalized to disordered proteins. This is because even though the folded 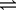 unfolded equilibrium is not well defined, similar to well structured proteins, disordered proteins also exist either in a soluble monomeric (instead of the folded state), a misfolded/aggregated, and a complexed state. Many disordered proteins acquire a definite structure when bound to their interaction partners and seldom dissociate to the soluble monomeric [31]. These serve as even stronger candidates for the beneficiaries of interaction-induced stability compared to folded proteins. Consequently, we include both partially disordered proteins and structured proteins in the current analysis of the 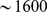 cytoplasmic proteins.

### Suggested experimental tests

#### Modulation of protein stability by overexpression of its partners

We predict that the measured free energy of protein folding in vivo [32], [33] will be lower than the in vitro measurement. Moreover, this free energy can be modulated by overexpressing the interaction partners of the protein that increases the equilibrium constant 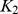 between the folded monomer and the generic complexed state. Recently, it was observed that the measured stability of phosphoglycerate kinase was higher by 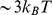 *in vivo* compared to *in vitro* [33].

#### Overexpression-instability epistasis

Does the PPI-induced stabilization have evolutionary advantages? We propose the following experimental test. Consider two mutated phenotypes for an isolated interacting pair of proteins A and B in an organism 1) 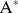, a destabilized mutant of protein A and 2) 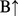 where B is overexpressed. We predict that lowering of the organismal fitness due to destabilization of protein A (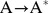) can be at least partially rescued by the overexpression of the protein B (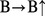) i.e. the combination of two penalizing mutations may perhaps be advantageous to the organism.

## Methods

### Law of mass action and 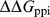


**Figure 5:**
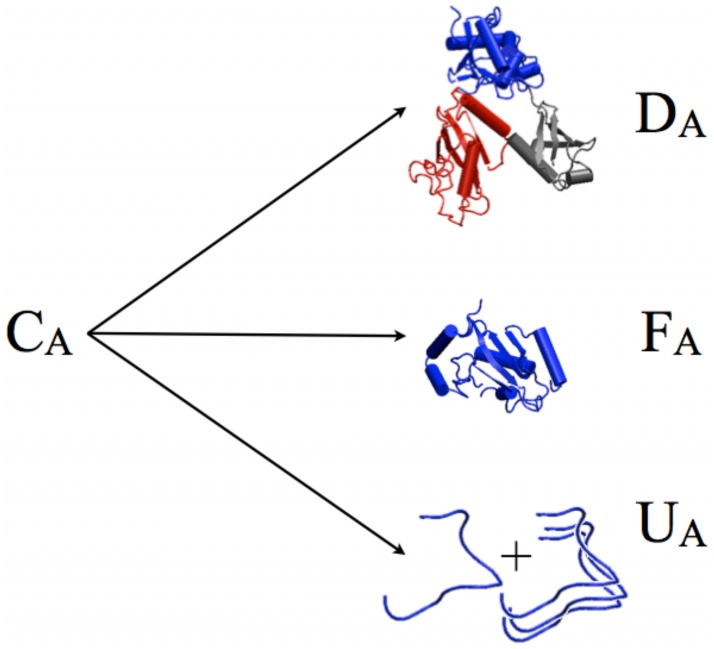
At steady state, protein A can be present either as a mixture of misfolded monomers and insoluble oligomers (U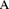), a folded monomer F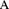, or in a complex with its interaction partners (D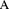).

In cellular homeostasis, the total concentration 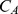 of any protein 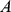 can be written as the sum of its free folded monomer concentration 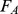, a fraction comprising of insoluble oligomers and unfolded peptide 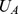, and as part of all protein complexes 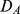 containing 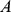 (See Fig. 5). In our computational model, for simplicity and owing to the nature of the large scale data [34], we restrict protein complexes to dimers [35], thus for all proteins 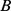 that interact with 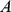,
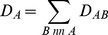
(2)Conservation of mass implies,
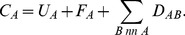
(3)The concentration 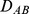 of each dimer 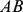 satisfies the law of mass action,
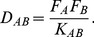
(4)We can write the balance between the three states of the protein, 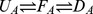 (See Fig. 1), as two *equilibrium* equations
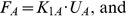
(5)

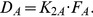
(6)Note that 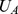 comprises of a collection of biologically unusable states of the protein viz. the misfolded/unfolded and the oligomerized state any of which may convert to/interact with the folded monomeric state 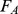. Consequently, the first equilibrium 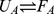 is a collection of *thermodynamic equilibriums*. The equilibrium constant 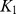 will thus depend not only on the temperature 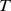 but also on 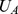 and 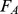. If among the unfolded, misfolded, and the oligomerized states the former dominates the population comprising 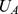 then, 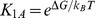 where 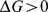 is the thermodynamic stability of the free monomeric state. Similarly, 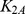 is given by,
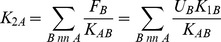
(7)and depends not only on the dissociation constants 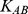 but also the free concentrations 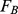 of the interacting partners of protein 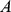 and on the topology of the interaction network in the organism. Here too, we assume that a) only the folded monomeric forms of proteins interact with each other and b) there is no appreciable interaction between the collective unfolded state 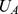 of protein 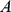 and any state of any other protein 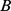. We have also neglected the role of chaperones in actively reducing the concentration of the unfolded/misfolded/aggregated state by turning it over to the folded state. In fact, some of the chaperones are included in of our mass action equilibrium model and prevent unfolding by sequestering the folded state (see below and the discussion section).

By combining mass conservation (Eq. 3) with Eq. 5 and Eq. 6,
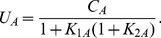
(8)In the above development, we have made a crucial assumption that only.

Note that in the absence of interactions, 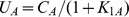. We identify 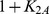 as the additional decrease in the insoluble fraction due to protein-protein interactions. We define the interaction-induced stability 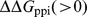 as,
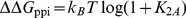
(9)


### Identification of proteins and the mass action model

We downloaded the latest set of interacting proteins in baker's yeast from the BIOGRID database [36]. To filter for non-reproducible interactions and experimental artifacts, we retained only those interactions that were confirmed in two or more separate experiments. For the sake of simplicity, we only considered cytoplasmic proteins [37] with known concentrations [38]. This lead to 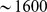 proteins connected by 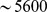 interactions.

The *in vivo* stability of a protein is a combination of its thermodynamic stability, resistance to aggregation or oligomerization, and resistance to degradation [39]. Note that the interaction-induced stability of a protein depends on the stability of its interaction partners (see Eq. 6, Eq. 7, and Eq. 9). Unfortunately, the exact dependence of the *in vivo* protein stability on its sequence is unclear and there exist no reliable data or sequence dependent computational estimates for the thermodynamic stability of proteins. Moreover, 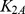, and thus 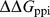 (Eq. 6, Eq. 7, and Eq. 9), can be estimated even in the absence of the knowledge of 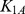. In our estimates of 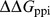, we assume that 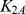 is given simply by
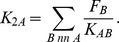
Here, 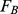 is obtained by solving the mass action equations [35] iteratively (see below). This is equivalent to assuming that all the proteins are equally and highly stable (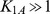 for all proteins 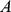). The 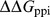 thus calculated serves as the upper limit of interaction-induced stability. In the supplementary materials (Text S1, Fig. S1, Fig. S2, and Tables S4 and S5), we show that different assignments of the equilibrium constants including a simple model of protein stability [40]–[42] do not change the qualitative nature of our observations.

The dissociation constants 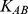 for protein-protein interactions follow a lognormal distribution with a mean 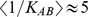 nM [35]. The majority of interactions between proteins are neither too weak nor unnecessarily strong. Common sense dictates that it does not make sense to decrease the dissociation constant between two proteins beyond the point where the abundance limiting protein spends all of its time in the bound state. Motivated by these evolutionary arguments to minimize unnecessary protein production and to avoid unnecessarily strong interactions, Maslov and Ispolatov [35] devised a recipe to assign dissociation constants to individual protein-protein interactions. viz. for interacting proteins 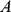 and 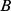, the dissociation constant 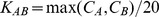. We also explore a few other assignment rules for dissociation constants (see supplementary Text S1, Fig. S3, and Table S6).

We solve for free concentrations 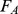 iteratively [35]. We start by setting 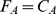 for all proteins and iteratively calculate 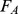 from
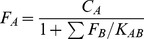
(10)till two consecutive estimates of 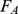 fall within 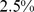 of each other for all proteins.

### Simplified fitness model for cellular proteomes

As noted above, the toxic effects of misfolding and aggregation may be the chief determinant of protein sequence evolution [2], [4], [5]. The dosage dependent fitness effect of misfolded proteins [20] motivates us to introduce a simple biophysical model for fitness 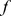 of the proteome (See Eq. 11),
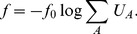
(11)

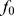
 is the scaling factor. Potentially, 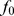 can be estimated from fitness experiments by introducing measured quantities of unfolded protein in the cell [20]. We explore the evolution of a hypothetical proteome to investigate the interplay between protein stability and protein-protein interactions.

We believe that protein abundances and the topology of the interaction network are largely dictated by biological function. It is non-trivial to incorporate the fitness effect of changes in gene expression level and the network topology in our simplified model. Thus, to specifically probe the relation between stability and interactions, we concentrate on the effect of toxic gain of function due to misfolding and aggregation on cellular fitness and not include changes in gene expression levels and network topology. In this aspect, our model is in the same spirit as previously proposed models [6], [41]–[48]. The effect of random mutations on average destabilizes proteins and the dynamics of the evolution of thermodynamic stability of proteins can be modeled as a random walk with negative average velocity [6]. We consider the thermodynamic stability as a proxy for the *in vivo* stability of proteins. We construct the cytoplasm of a hypothetical organism with 15 proteins. The number of proteins is low due to computational restrictions. The proteome is evolved by sampling the dissociation constants from the lognormal distribution while introducing random mutations in proteins that change their stability. At each generation, the fitness is evaluated and the progeny is accepted at a certain *evolutionary temperature* (defined as the inverse of the effective population size, 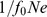) [21]. We run a total of 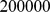 generations for each *evolutionary temperature* and analyze the organism in the latter half of the evolutionary run (details of the model and a brief description of the population genetics terminology is in supplementary Text S1).

### Aggregation propensity

The notion of protein stability relevant to this study is the propensity of a protein to avoid structural transformations that may render it unemployable for biological function. For example, for a small and highly soluble protein, this stability corresponds to the thermodynamic stability of the native state while for a large multi domain protein, it may correspond to the thermodynamic stability of one of its domains against the partially unfolded state. In short, thermodynamic stability of the folded state with respect to the unfolded, partially folded state, and the misfolded state all contribute to the *in vivo* stability of proteins [39].

Though there is a lack of proteome-wide estimates of thermodynamic stability of proteins, the aggregation propensity can be estimated from the sequence [24], [26] and is known to be correlated with protein stability [24]. In our correlation analysis, we use the estimated aggregation propensity as a proxy for *in vivo* protein stability and explore the relationship between interaction-induced stability 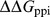 and protein stability. The aggregation propensity was estimated for the same 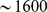 proteins used in the mass action calculation to estimate 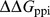. We tested the TANGO [24] and Aggrescan [26] to estimate the aggregation propensity of proteins. Previously, TANGO has been used [22], [23], [49] to understand the relation between protein abundance and instability. We show results for TANGO in the main text. Aggrescan results (supplementary Text S1 and Table S3) are quite similar.

## Supporting Information

Figure S1The histogram of interaction-induced stabilities 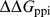 when protein stabilities depend on their chain length.(TIF)

Figure S2The histogram of interaction-induced stabilities 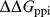 when protein stabilities are set at their minimum.(TIF)

Figure S3The histogram of interaction-induced stabilities 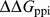 when all dissociation constants are set at 5 nM.(TIF)

Figure S4 The histogram of estimated disorder in the proteins of the yeast proteome.
(TIF)

Table S1 A table for the parameters and topology of the toy proteome.
(PDF)

Table S2 A table reporting correlations between stability and interaction using TANGO [24].
(PDF)

Table S3 A table reporting correlations between stability and interaction using AGGRESCAN [26].
(PDF)

Table S4 A table reporting correlations between stability and interaction when protein stabilities depend on their chain length.
(PDF)

Table S5 A table reporting correlations between stability and interaction when protein stabilities are set to their minumum.
(PDF)

Table S6 A table reporting correlations between stability and interaction when all dissociation constants are set at 5 nM.
(PDF)

Text S1 An inventory of population genetics terms, additional information about the toy model, and misc. information about the analysis.
(PDF)
