# Supplementary figures and images for: Evolutionary Capacitance and Control of Protein Stability in Protein-Protein Interaction Networks
Source: PLoS Comput Biol. 2013 Apr 4;9(4):e1003023. doi: 10.1371/journal.pcbi.1003023 (PMC3617028; doi:10.1371/journal.pcbi.1003023)

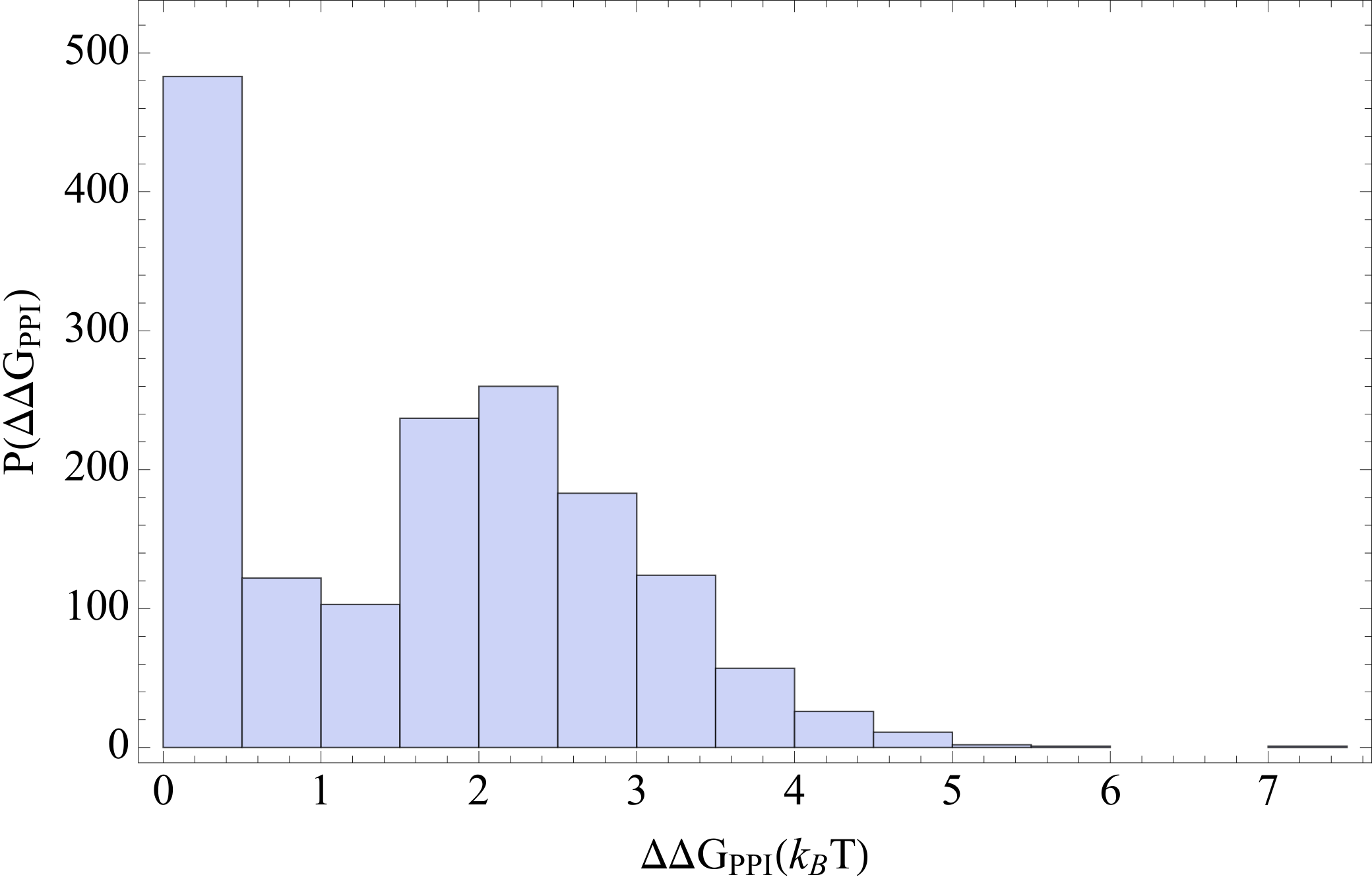

Supplement: Figure S1 — The histogram of interaction-induced stabilities when protein stabilities depend on their chain length. (TIF) [file pcbi.1003023.s001.tif]

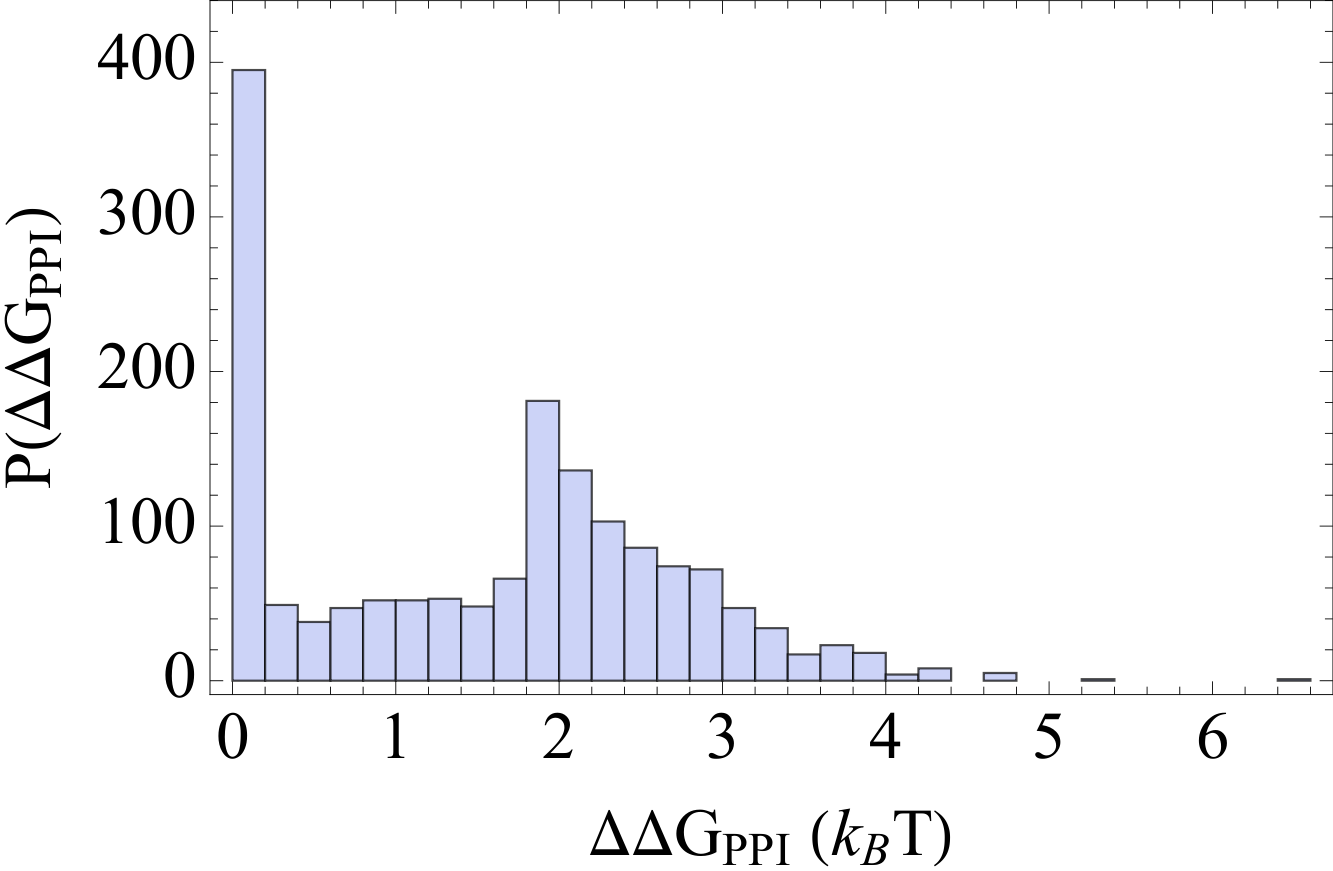

Supplement: Figure S2 — The histogram of interaction-induced stabilities when protein stabilities are set at their minimum. (TIF) [file pcbi.1003023.s002.tif]

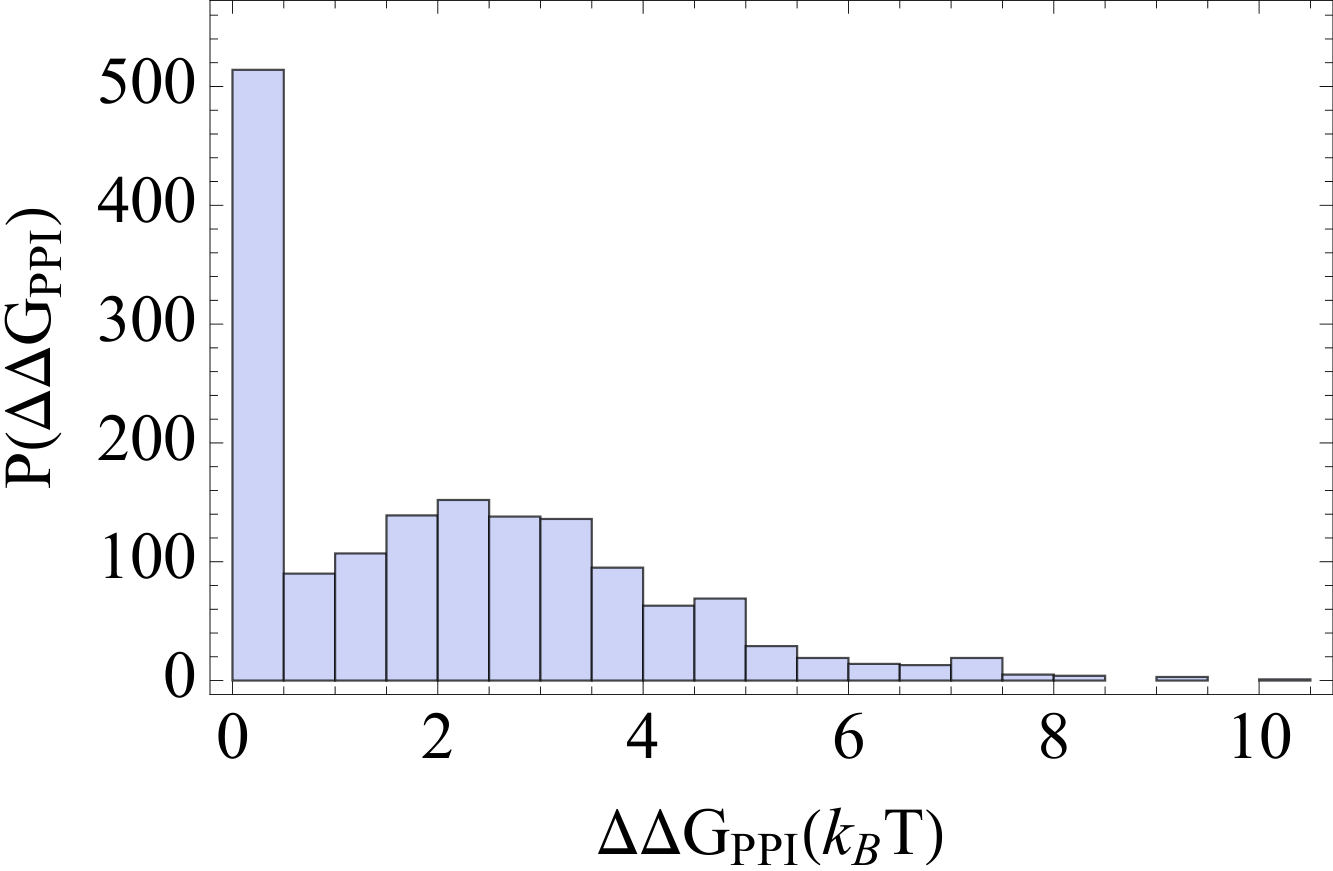

Supplement: Figure S3 — The histogram of interaction-induced stabilities when all dissociation constants are set at 5 nM. (TIF) [file pcbi.1003023.s003.tif]

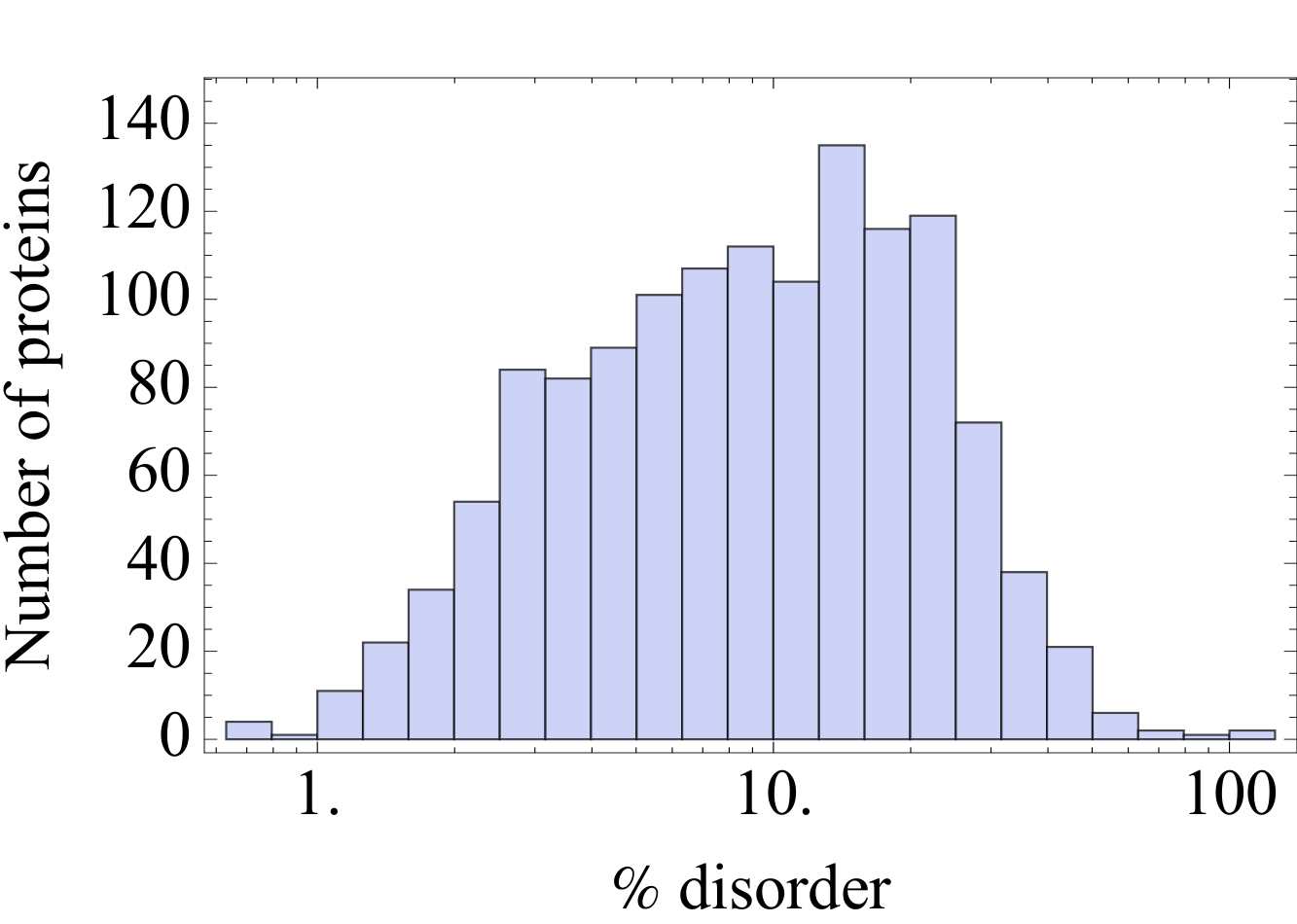

Supplement: Figure S4 — The histogram of estimated disorder in the proteins of the yeast proteome. (TIF) [file pcbi.1003023.s004.tif]
