# Supplementary material for: Evolutionary Capacitance and Control of Protein Stability in Protein-Protein Interaction Networks
Source: PLoS Comput Biol. 2013 Apr 4;9(4):e1003023. doi: 10.1371/journal.pcbi.1003023 (PMC3617028; doi:10.1371/journal.pcbi.1003023)
Supplement: Table S1 — A table for the parameters and topology of the toy proteome. (PDF) [file pcbi.1003023.s005.pdf]

Protein	Concentration (nM)	Interactions
1	9.05	1, 2, 3, 4
2	510.16	1, 5
3	259.61	1
4	70.33	1
5	51.02	2
6	3048.92	7, 11
7	221.27	6
8	513.18	-
9	609.78	-
10	16.25	12, 13
11	38.33	6, 15
12	624.82	10, 13
13	655.06	10, 12
14	670.15	-
15	7.81	11

TABLE S1. A Table of concentrations and interaction partners of proteins in the simplified proteome. The concentrations are chosen from the yeast proteome.
