# Supplementary material for: Evolutionary Capacitance and Control of Protein Stability in Protein-Protein Interaction Networks
Source: PLoS Comput Biol. 2013 Apr 4;9(4):e1003023. doi: 10.1371/journal.pcbi.1003023 (PMC3617028; doi:10.1371/journal.pcbi.1003023)
Supplement: Table S2 — A table reporting correlations between stability and interaction using TANGO [24]. (PDF) [file pcbi.1003023.s006.pdf]

	Aggregation	Control variables $\Delta\Delta G_{PPI}$	C	F
$\Delta\Delta G_{PPI}$	0.11*	-	0.11*	0.02
C	-0.11*	-0.10*	-	-0.03
F	-0.15*	-0.11*	-0.10*	-

TABLE S2: The second column reports the spearman rank correlation between the TANGO aggregation propensity and $\Delta\Delta G_{PPI}$, total protein concentration *C*, and LMA estimated free monomer concentration *F*. The Asterix implies p < 10^{-3}. The third column reports the partial correlation between aggregation propensity and *C* and *F* when $\Delta\Delta G_{PPI}$ is controlled. The fourth column reports the partial correlation between aggregation propensity and $\Delta\Delta G_{PPI}$ and *F* when *C* is controlled and finally, the fifth column reports the partial correlation between aggregation propensity and $\Delta\Delta G_{PPI}$ and *C* when *F* is controlled. Note, the correlation between aggregation propensity and estimated free monomer concentration is statistically significant even after controlling for total concentration.
