# Supplementary material for: Evolutionary Capacitance and Control of Protein Stability in Protein-Protein Interaction Networks
Source: PLoS Comput Biol. 2013 Apr 4;9(4):e1003023. doi: 10.1371/journal.pcbi.1003023 (PMC3617028; doi:10.1371/journal.pcbi.1003023)
Supplement: Table S3 — A table reporting correlations between stability and interaction using AGGRESCAN [26]. (PDF) [file pcbi.1003023.s007.pdf]

	Aggregation	Control variables $\Delta\Delta G_{PPI}$	C	F
$\Delta\Delta G_{PPI}$	0.12^{*}	-	0.13^{*}	0.02
C	-0.11^{*}	-0.14^{*}	-	-0.004
F	-0.17^{*}	-0.12^{*}	-0.11^{*}	-

TABLE S3: Analysis similar to **Table S2**, replacing TANGO (5) with AGGRESCAN (4).
