# Supplementary material for: Evolutionary Capacitance and Control of Protein Stability in Protein-Protein Interaction Networks
Source: PLoS Comput Biol. 2013 Apr 4;9(4):e1003023. doi: 10.1371/journal.pcbi.1003023 (PMC3617028; doi:10.1371/journal.pcbi.1003023)
Supplement: Table S4 — A table reporting correlations between stability and interaction when protein stabilities depend on their chain length. (PDF) [file pcbi.1003023.s008.pdf]

	Aggregation	Control variables $\Delta\Delta G_{PPI}$	C	F
$\Delta\Delta G_{PPI}$	0.08*	-	0.08*	-0.007
C	-0.11*	-0.11*	-	0.007
F	-0.15*	-0.12*	-0.10*	-

TABLE S4: Analysis similar to **Table S2** when protein stabilities are solely dependent on the chain length.
