# Supplementary material for: Evolutionary Capacitance and Control of Protein Stability in Protein-Protein Interaction Networks
Source: PLoS Comput Biol. 2013 Apr 4;9(4):e1003023. doi: 10.1371/journal.pcbi.1003023 (PMC3617028; doi:10.1371/journal.pcbi.1003023)
Supplement: Table S5 — A table reporting correlations between stability and interaction when protein stabilities are set to their minumum. (PDF) [file pcbi.1003023.s009.pdf]

	Aggregation	Control variables		
		$\Delta\Delta G_{PPI}$	C	F
$\Delta\Delta G_{PPI}$	0.11*	-	0.11*	0.02
C	-0.11*	-0.11*	-	-0.02
F	-0.14*	-0.10*	-0.10*	-

TABLE S5: Analysis similar to **Table S2** when the stabilities of all proteins are set to their minimum $\Delta G_{\text{folding}} = 0$.
