# Supplementary material for: Evolutionary Capacitance and Control of Protein Stability in Protein-Protein Interaction Networks
Source: PLoS Comput Biol. 2013 Apr 4;9(4):e1003023. doi: 10.1371/journal.pcbi.1003023 (PMC3617028; doi:10.1371/journal.pcbi.1003023)
Supplement: Table S6 — A table reporting correlations between stability and interaction when all dissociation constants are set at 5 nM. (PDF) [file pcbi.1003023.s010.pdf]

	Aggregation	Control variables $\Delta\Delta G_{PPI}$	C	F
$\Delta\Delta G_{PPI}$	0.08*	-	0.10*	0.02
C	-0.11*	-0.13*	-	0.004
F	-0.17*	-0.13*	-0.11*	-

TABLE S6: Analysis similar to **Table S2** when the dissociation constants for all protein-protein interactions are set at K_{AB} = 5nM.
