# Supplementary material for: Evolutionary Capacitance and Control of Protein Stability in Protein-Protein Interaction Networks
Source: PLoS Comput Biol. 2013 Apr 4;9(4):e1003023. doi: 10.1371/journal.pcbi.1003023 (PMC3617028; doi:10.1371/journal.pcbi.1003023)
Supplement: Text S1 — An inventory of population genetics terms, additional information about the toy model, and misc. information about the analysis. (PDF) [file pcbi.1003023.s011.pdf]

I. SUPPLEMENTARY MATERIALS

A. Details of the simplified proteome

There are 15 proteins in the simplified proteome. Table S1 has the concentrations and interaction partners of all 15 proteins. The concentrations were chosen from the yeast proteome.

Random mutations typically make proteins unstable. In the monte carlo simulation of the simplified proteome, the evolution of protein stability is modeled as a random walk with a drift towards instability (1). We do not allow the free energy of the folded state to fall below 0 (the folded state is always at least as stable as the unfolded state). The protein-protein interaction energies are chosen from a normal distribution (a lognormal distribution for dissociation constants) with mean $\mu = 10\ k_BT$ and deviation $\sigma = 2.5\ k_BT$. The statistical correlations do not depend on the specific value of the mean and the standard deviation. Each step of the monte carlo simulation allows some of the proteins to change their stability and interaction strengths. The *fitness* of the proteome is determined by the total unfolded protein concentration. The monte carlo step is accepted at a particular *evolutionary temperature* according to the Metropolis criterion.

B. A brief description of population genetics terms

We briefly describe the population genetics terms used in the main text. We refer the readers to classic textbooks such as (2) and a recent paper (3) for more details.

- The effective population size Ne can be understood as *the number of individuals in an idealized population that would show the same amount of dispersion of allele frequencies under random genetic drift as the population under consideration.* The effective population is strictly less than the actual population (the consensus population).

- Evolutionary biology understands the fitness f of a genotype as the propensity of that genotype to propagate to the next generation. In a population of fixed size with individuals with genotypes of different fitness, those with a genotype of higher fitness are more likely to be present in the next generation.

- If the population size Ne is small, due to random chance, genotypes with suboptimal fitness may be carried to the next generation in a process known as random genetic drift. The degree of suboptimality that is likely to propagate depends on the effective population size. In the canonical ensemble picture of evolution where effective population scales as inverse temperature (3), suboptimality corresponds to an increase in *thermal fluctuations* at higher evolutionary temperatures (low effective population) compared to lower temperatures (high effective populations).

C. Table for the statistical analysis with TANGO

We collect the various Spearman and partial Spearman correlation coefficients in Table S2.

D. Statistical analysis for Aggrescan (4)

We use the *area above hotspot threshold* or AAHT as an estimator of aggregation propensity (4). The area estimates the region of the protein sequence that is deemed aggregation prone by the Aggrescan (4) algorithm. AAHT correlates well with the aggregation propensity predicted by TANGO ($p < 10^{-5}$). Similar to TANGO, AAHT correlates negatively with concentration (Spearman $r = -0.13\ p < 10^{-5}$) and with the free monomer concentration (Spearman $r = -0.17\ p < 10^{-5}$).

In Table S3, we reproduce the observed correlations (similar to Table S2) with AAHT as a predictor of aggregation propensity.

Similar to the TANGO estimate, the effect of protein knockout or the capacitance $\mathcal{C}_i$ of protein i (see Discussion in the main text) also correlates with $\langle Z_{\mathrm{neighbors}} \rangle$, the average aggregation propensity of its neighbors (Spearman $r = 0.53,\ p < 10^{-5}$) even after controlling for protein abundance (Spearman $r = 0.59,\ p < 10^{-5}$).

E. Sequence length dependent models (6–8) do not change our conclusions

Due to the lack of sequence dependent estimators of protein stability, while calculating the interaction-induced stability (See Eq. 8 in main text), we assumed that all proteins are infinitely stable i.e. $K_1 \to \infty$ in Eq. 7 in main text. The estimates of interaction-induced stability therein are thus the upper limit of protein stability.

Here, we show that employing a sequence length dependent model (6–8) of stability does not change our conclusions. In Figure S1, we plot the histogram of induced stabilities $\Delta\Delta G_{\mathrm{ppi}}$ by taking into account the finite stability of proteins as predicted by their sequence length. Observe that the distribution of induced stabilities is very similar to the one observed when all proteins are infinitely stable (See Fig. 3 in main text).

Moreover, the correlations observed in Table S2 are also reproduced, albeit slightly weakly yet statistically significantly (See Table S4), when we take into account protein stabilities.

F. Setting all $K_{1A} = 1$ does not change our conclusions

We also set the folding free energy $\Delta G_{\text{folding}}$ to 0 i.e. $K_{1A} = 1$ for all proteins and estimate the interaction induced stability. In Figure S2, we plot the histogram of induced stabilities $\Delta\Delta G_{\text{ppi}}$ when $\Delta G_{\text{folding}} = 0$ for all proteins. Observe that the distribution of induced stabilities is very similar to the one observed when all proteins are infinitely stable (See Fig. 3 in main text).

The correlations observed in Table S2 are also reproduced statistically significantly (See Table S5), when protein stabilities are set to their minimum.

G. Alternative assignment of interaction constants does not change our conclusions

In our estimates of interaction-induced stability, similar to Maslov and Ispolatov (9), we assign the dissociation constant K_{ij} for two proteins i and j with concentrations C_i and C_j respectively by $K_{ij} = \frac{max(C_i,C_j)}{20}$. The above assignment minimizes the free monomer concentration(s) while reproducing the overall distribution of experimentally known dissociation constants.

In Figure S3, we show the histogram of interaction-induced stabilities when all dissociation constants are set at their estimated average $K_{ij} = 5$ nM (9). Note that the stabilities are slightly higher than in the main text. A similar analysis at $K_{ij} = 10$ nM also reproduces the qualitative conclusions (not shown). Moreover as seen in Table S6, the correlations observed in Table S2 are reproduced as well.

We also test the robustness of the predicted histogram of interaction-induced stabilities by shuffling the dissociation constants between interacting proteins. We find that 50 different realizations of dissociation constants shuffled on the interaction network robustly reproduce the mean ($\langle\Delta\Delta G_{\text{ppi}}\rangle \approx 2.3\ k_BT$) and the variance ($\langle\delta\Delta\Delta G^2_{\text{ppi}}\rangle \approx 8.8\ k_BT^2$) of the $P(\Delta\Delta G_{\text{ppi}})$, the distribution of interaction-induced stabilization.

H. Disordered proteins

The proteins in higher organisms have stretches of disordered regions in them. Even though our development strictly applies only to proteins with a well defined folded and unfolded states, it can be easily generalized to proteins with partial disorder. Similar to folded proteins, partially disordered proteins too have a soluble monomeric state and an insoluble oligomerized state; even though the transition between the folded and the unfolded state may not be well defined. Moreover, many disordered regions in proteins acquire a three dimensional structure after binding to their interaction partners. Here, we present the estimates of disordered regions in the analyzed proteome of yeast ($\sim$ 1600 cytoplasmic proteins). We use the freely available DisEMBL program (10).

Figure S4 shows the histogram of estimated % disordered amino acids for the $\sim$ 1600 proteins considered in this study. We find that on an average 10% of the amino acids in a given protein are disordered.

I. List of capacitors

The CSV file contains a list of top 20 capacitors identified in the current study.

J. Captions for supplementary figures

1. The histogram of estimated ppi-induced stabilities for the yeast cytoplasmic proteome when the inherent stability of proteins is modeled solely on the basis of their chain length (6–8). Similar to Fig. 3 in main text, the average stability is ~ 2 k$_B$T and some proteins can receive as much as $5-6$ k$_B$T of stability from their binding partners.

2. The histogram of estimated ppi-induced stabilities, similar to Figure S1, when all protein stabilities are set at their minimum $\Delta G_{\text{folding}} = 0$ or $K_{1A} = 1$.

3. The histogram of estimated ppi-induced stabilities, similar to Figure S1, when the dissociation constants for all protein-protein interactions are set at $K_{AB} = 5$ nM.

4. The histogram of the estimated % of amino acids in a disordered state (10) in a protein for proteins considered in this analysis. For majority of the proteins, $\sim$ 10% of the amino acids are in a disordered state. Note that there are a very few completely disordered proteins.

[1] Zeldovich, K. B., P. Chen, and E. I. Shakhnovich, 2007. Protein stability imposes limits on organism complexity and speed of molecular evolution. *Proc. Natl. Acad. Sci.* 104:16152–16157.
[2] Fisher, R. A., 1930. The genetical theory of natural selection. Clarendon Press, Oxford, England.
[3] Sella, G., and A. E. Hirsh, 2005. The application of statistical physics to evolutionary biology. *Proc. Natl. Acad. Sci.* 102:9541–9546.

[4] Conchillo-Sole, O., N. S. de Groot, F. X. Aviles, J. Vendrell, X. DAura, and S. Ventura, 2007. AGGRESCAN: a server for prediction and evaluation of “hot spots” of aggregation in polypeptides. *Bioinfo.* 8:65.

[5] Fernandez-Escamilla, A. M., J. Schymkowitz, and L. Serrano, 2004. Prediction of sequence-dependent and mutational effects on the aggregation of peptides and proteins. *Nature Biotech.* 22:1302–1306.

[6] Ghosh, K., and K. A. Dill, 2009. Computing protein stabilities from their chain lengths. *Proc. Natl. Acad. Sci.* 106:10649–10654.

[7] Dill, K. A., K. Ghosh, and J. D. Schmit, 2011. Physical limits of cells and proteomes. *Proc. Natl. Acad. Sci.* 108:17876–17882.

[8] Ghosh, K., and K. A. Dill, 2010. Cellular proteomes have broad distributions of protein stability. *Biophys. J.* 99:3996–4002.

[9] Maslov, S., and I. Ispolatov, 2007. Propagation of large concentration changes in reversible protein-binding networks. *Proc. Natl. Acad. Sci.* 104:13655–13660.

[10] Linding, R., L. Jensen, F. Diella, P. Bork, T. Gibson, R. Russell, et al., 2003. Protein Disorder Prediction-Implications for Structural Proteomics. *Structure* 11:1453.
